# The Anti-*Pseudomonas aeruginosa* Antibody Panobacumab Is Efficacious on Acute Pneumonia in Neutropenic Mice and Has Additive Effects with Meropenem

**DOI:** 10.1371/journal.pone.0073396

**Published:** 2013-09-02

**Authors:** Thomas Secher, Stefanie Fas, Louis Fauconnier, Marieke Mathieu, Oliver Rutschi, Bernhard Ryffel, Michael Rudolf

**Affiliations:** 1 Université d’Orléans and Centre National de la Recherche Scientifique, Unité Mixte de Recherche, Orléans, France; 2 Kenta Biotech AG, Schlieren, Switzerland; 3 Institute of Infectious Disease and Molecular Medicine, University of Cape Town, Cape Town, Republic of South Africa; University of Iowa Carver College of Medicine, United States of America

## Abstract

*Pseudomonas aeruginosa* (*P. aeruginosa*) infections are associated with considerable morbidity and mortality in immunocompromised patients due to antibiotic resistance. Therefore, we investigated the efficacy of the anti-*P. aeruginosa* serotype O11 lipopolysaccharide monoclonal antibody Panobacumab in a clinically relevant murine model of neutropenia induced by cyclophosphamide and in combination with meropenem in susceptible and meropenem resistant *P. aeruginosa* induced pneumonia. We observed that *P. aeruginosa* induced pneumonia was dramatically increased in neutropenic mice compared to immunocompetent mice. First, Panobacumab significantly reduced lung inflammation and enhanced bacterial clearance from the lung of neutropenic host. Secondly, combination of Panobacumab and meropenem had an additive effect. Third, Panobacumab retained activity on a meropenem resistant *P. aeruginosa* strain. In conclusion, the present data established that Panobacumab contributes to the clearance of *P. aeruginosa* in neutropenic hosts as well as in combination with antibiotics in immunocompetent hosts. This suggests beneficial effects of co-treatment even in immunocompromised individuals, suffering most of the morbidity and mortality of *P. aeruginosa* infections.

## Introduction

*P. aeruginosa* is a virulent pathogen leading to a broad range of acute and chronic infections. In particular, nosocomial lung infections are associated with high morbidity and mortality in immunocompromised patients, e.g. after organ transplantation, severe burn, cancer, HIV infection and neutropenic patients [1,2]. Infections in this patient population are problematic, partly due to the immunocompromised status that allows bacteria to spread systemically and to resist to antibiotics. The classical antibiotics with antipseudomonal activities include aminoglycosides, ceftazidime and carbapenems. Although carbapenems have been shown to be effective in nosocomial pneumonia and are currently the most prescribed antibiotics, 15% of *P. aeruginosa* clinical isolates are resistant to imipenem [3]. Imipenem has been shown to be associated with frequent development of drug resistance of *P. aeruginosa* [4], inducing the exacerbation of the host inflammatory response, as shown for ceftazidime [5].

Its large genome predisposes *P. aeruginosa* to survive in a hostile environment. In addition to an array of exo/endotoxins and enzymatic products that hijack host defense, *P. aeruginosa* harbors both chromosomal and/or plasmid encoded antibiotic resistance genes, limiting antibiotic treatment efficacy [6]. Indeed, it has been established that *P. aeruginosa* is the most common multidrug-resistant (MDR) gram-negative pathogen causing pneumonia in hospitalized patients [7]. Recent reports includes *P. aeruginosa* in the group of ESKAPE pathogens which exhibited resistance to all available drugs [8], even to colistin [9]. The overall clinical data reveal significant antibiotic resistance development over the last 15 years. In addition to the reduction of therapeutic options, antibiotic resistance to *P. aeruginosa* has an increasing impact on patient mortality and hospitalization cost. In fact, studies have highlighted that 67% of patient mortality was associated with resistant strains [10], along with a significant increase of median hospital stay and costs [11].

Therefore, treatment of *P. aeruginosa* remains a challenge and a high medical need exists for novel therapeutic approaches, especially in high risk patient populations. The increased prevalence of MDR strains has led to the emergence of new experimental anti-pseudomonas agents besides antibiotics. Antibody immunotherapy may be a valuable addition to standard antibiotic therapy against pneumonia. Nevertheless, only a few groups have reported positive experimental data using anti-LPS [12], anti-flagella [13] or anti-PcrV [14,15] antibodies against *P. aeruginosa* infection with limited clinical data. Recently, a small phase IIa study of the fully human IgM antibody Panobacumab was successfully completed in hospital acquired pneumonia patients, suggesting a potential therapeutic impact of Panobacumab treatment [16]. Panobacumab is an IgM/κ monoclonal antibody directed against the LPS O-polysaccharide moiety of *P. aeruginosa* serotype IATS O11. It has been recently characterized *in vitro* [17] and its safety and efficacy has been demonstrated in mice [18] and humans [19].

However, the clinical situation differs from the experimental conditions, e.g. in patients an antibody will always be administered in combination with antibiotics. So far, no preclinical evidence for the efficacy of Panobacumab therapy in models of immunosuppression has been established, yet a substantial number of ICU patients are immunosuppressed. Thus, there is a clear need for a proof of concept of the efficacy of antibody treatment in *in vivo* mouse models mimicking these clinical conditions.

Here, we investigate the efficacy of Panobacumab treatment in various experimental models of *P. aeruginosa* that mimic clinical settings encountered among *P. aeruginosa*-infected patients. We study the effect of panobocumab in leukopenic mice representing a model of neutropenia. We report that treatment with Panobacumab resulted in enhanced bacterial clearance with subsequent attenuated lung inflammation in the context of acute neutropenia. In addition, we studied the effect of Panobacumab in the presence of Meropenem, a prototypal antibiotic primarily administered after detection of a *P. aeruginosa* infection in a clinical situation. We observe an additive effect of Panobacumab with Meropenem on bacterial load and pneumonia as compared to single agent treatment. Moreover panobacumab-induced reduction of acute pneumonia is still effective in Meropenem-resistant *P. aeruginosa* pneumonia.

## Materials and Methods

### Mice

Female C57BL/6 (B6) mice of 10-12 week of age were obtained from Janvier (Le Genest Saint-Isle, France). All mice were housed under specific pathogen-free conditions at the Transgenose Institute (Centre National de la Recherche Scientifique, Orléans, France) and had access to food and water *ad libitum*.

#### Ethic statement

All animal experiments complied with the French Government’s animal experiment regulations and were approved by the Ethics Committee for Animal Experimentation of the CNRS Campus of Orleans (CCO; N° CLE CCO 2011-026).

### Reagents

The generation of Panobacumab was previously described [13]. Panobacumab for injection was supplied as sterile, non-pyrogenic, phosphate-buffered saline (PBS) solution at a concentration of 312 µg/mL manufactured under GMP (Good Manufacturing Practice) condition (Kenta Biotech AG, Schlieren, Switzerland). The control antibody specific for *P. aeruginosa* serotype IATS-O1 was generated through the same technology (manuscript in preparation) and purified MAb was supplied by Kenta Biotech. Mice were injected intravenously with 0.4 mg/kg of Panobacumab in 200 µL of PBS, 4 h after infection. Control animals received PBS.

Meropenem was obtained from the pharmacy and reconstituted in saline at the concentration of 50 mg/mL. Aliquots of 1 ml were stored at -20°C and working solutions were prepared in isotonic saline. Mice were injected intraperitonealy with 30, 100 or 300 mg/kg in 200 µL of PBS, 2 hours after infection. Control animals received PBS.

### Bacterial strains

*P. aeruginosa* strains 2310.55 and 84, both serotype IATS O11, were provided by Kenta Biotech with certified titre and purity. Strain 2310.55 is a highly virulent clinical isolate from an urinary tract-infected patient, whereas strain 84 is a Meropenem resistant clinical isolate from a pneumonia patient (kind gift from Dr. Francois, Bruno, University of Limoges, France). Strain 84 was classified as Meropenem- resistant by disk diffusion assay (BD Sensi Disc 231704, Meropenem 10 mg). Uniformity of both strains was confirmed by plating on brain heart infusion (BHI) agar plates.

### Experimental model of neutropenia and *Pseudomonas aeruginosa* lung infection

For infections, an overnight culture in 10 mL BHI medium was prepared, starting from the frozen stock at 37°C and shaking at 150 rpm. 2.5mL of this culture was taken to start a fresh culture in 10 mL BHI. The culture was stopped when an OD of about 0.4 was reached (corresponding to a bacterial concentration of about 2x10^8^ bacteria/mL). The bacteria were washed once in PBS and diluted in saline to obtain a concentration of 10^6^ bacteria/40 µL. Each inoculum was then checked for accuracy by plating directly on fresh BHI agar plates. Mice were anesthetized i.v. with 200 µL with a low dose of Ketamine / Xylazine (1.25 mg/mL/0.5 mg/mL) and 40 µL of the bacterial solution or the corresponding vehicle solution (isotonic saline) was applied intranasally using an ultra fine pipette tip.

Neutropenia was induced by the intraperitoneal injection of cyclophosphamide (CP, Sigma) (50, 100, 200 mg/kg) [13] and evaluated 3, 5 or 7 days after CP injection. Neutropenic mice were infected with *P. aeruginosa*, 3 days after CP treatment, and neutropenic or non-neutropenic were all sacrificed 24 h after the infection.

### Hematologic analysis

Blood was drawn from mice, under anesthesia with isofluorane (CSP), into tubes containing EDTA (Vacutainer, Becton Dickinson), following manufacturer’s instructions. Hematologic parameters were determined using a 5-part-differential hematology analyzer (MS 9.5, Melet Schloesing Laboratoires).

### Broncho-alveolar lavage and organ sampling

Broncho-alveolar lavage fluid (BALF) was collected 24 h after *P. aeruginosa* administration by cannulating the trachea under deep ketamine/xylazine anaesthesia and washing the lung twice with 1 mL saline at room temperature. The lavage fluid was centrifuged at 2000 rpm for 10 min at 4°C and the supernatant was stored at -80°C for analysis. The cell pellet was resuspended in PBS, counted in a haemocytometer chamber and cytospin preparations were made using a Shandon cytocentrifuge (1000 rpm for 10 min). The cells were stained with Diff-Quick (Dade Behring, Marburg, Germany) and counted for neutrophils and macrophages.

### Lung bacterial load

Lung total weights were recorded after sacrifice and expressed as a percentage of body weight. Lung homogenates were prepared in 2 mL of isotonic saline solution using a Dispomix tissue homogenizer (Medic Tools). Ten-fold serial dilutions of homogenates were plated on brain-heart infusion agar plates (Biovalley). Plates were incubated at 37°C and 5% CO_2_, and the numbers of cfu were enumerated after 24 h.

### Cytokine determination

IL-6 and IL-1β concentrations in lung homogenates were measured by ELISA (Duoset Kit; R&D Systems) according to the manufacturer’s instructions (with detection limits at 50 pg/mL).

### Myeloperoxidase (MPO) activity

Lung homogenates were centrifuged at 10,000 _ *g* for 10 min at 4°C and the supernatant was discarded. The pellets were resuspended in 1 ml of PBS containing 0.5% hexadecyltrimethyl ammonium bromide (HTAB) and 5 mM EDTA and then incubated for 2 h at 60°C to inactivate the endogenous catalases. Following a new centrifugation, 50 µl of supernatant was placed in test tubes with 200 µl of PBS-HTAB-EDTA, 1.6 ml of HBSS, 100 µl of o-dianisidine dihydrochloride (1.25 mg/ml), and 100 µl of 0.05% H2O2 After 15 min of incubation at 37°C under agitation, the reaction was stopped with 100 µl of 1% NaN3. The MPO activity was determined as absorbance at 460 nm against blank (reaction mixture with saline in place of sample).

### Statistical analysis

Statistical evaluation of differences between the experimental groups was determined by using One-way ANOVA followed by a Tukey’ post test. All tests were performed with Graphpad Prism, Version 4.03 for Windows (GraphPad Software Inc., San Diego California USA, www.graphpad.com). All data are presented as mean ± Standard Error of the Mean (SEM). A p value < 0.05 was considered significant.

## Results

### *P. aeruginosa* clinical strain 2310.55, serotype O11 induces acute lung infection in neutropenic mice

**Figure 1:**
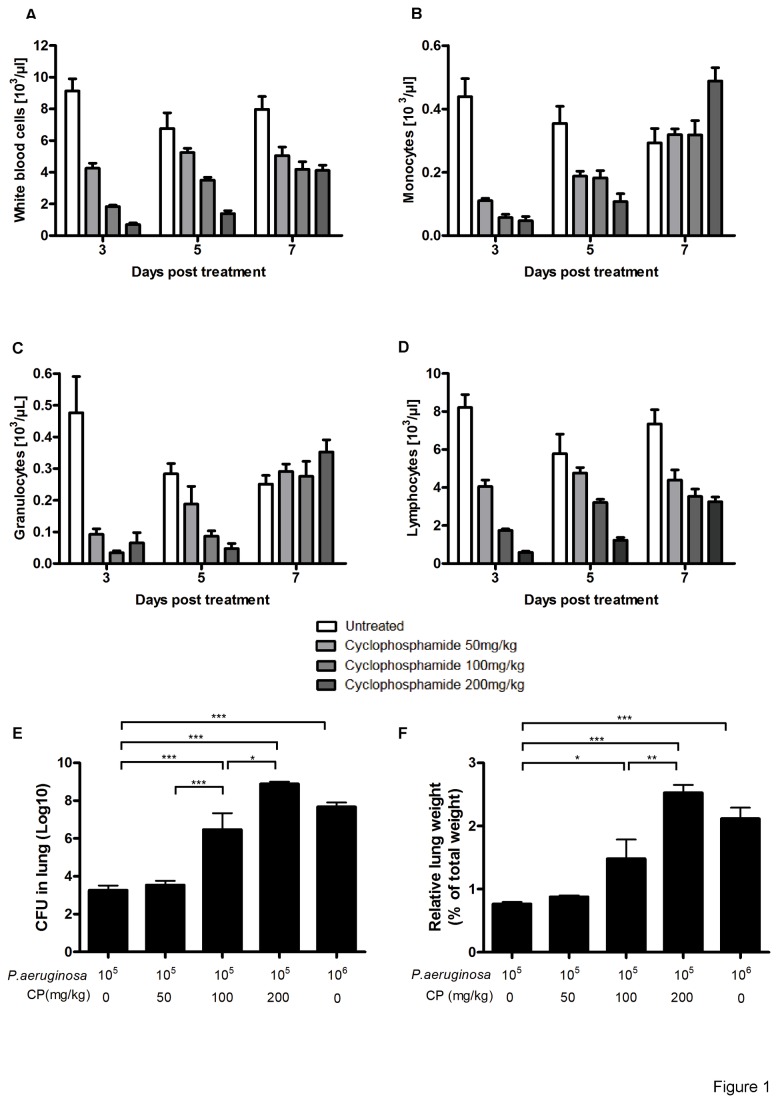
Enhanced *P. aeruginosa*-induced pneumonia in cyclophosphamide immunosuppressed mice. Myelodepression by cyclophosphamide causes neutropenia and enhanced pneumonia. Total white blood cells (A), monocytes (B), granulocytes (C) and lymphocytes (D) were determined. B6 mice received intra-peritoneal injection of 200 µL of cyclophosphamide at 50, 100 and 200 mg/kg and the hematogram was analyzed at 3, 5 and 7 days. Separate groups of mice were infected 3 days after CP injection by intra-nasal instillation of 40 µL of *P. aeruginosa* strain 2310.55 (10^5^ or 10^6^ cfu). Lung cfu (A) and lung weight (B) were recorded 24 h after infection. Groups of 7 mice were used and mean values ± SEM are shown (One-way ANOVA with Tukey’s Multiple Comparison Test; * p<0.05, ** p<0.01, *** p<0.001). The results are representative of three independent experiments.

Cyclophosphamide (CP), a well-known cytostatic and immunosuppressant drug, was used to induce neutropenia. We first established a dose and time dependency of CP in B6 mice to reduce circulating leukocytes in the blood. Significant and dose-dependent leucopenia was induced 3 days after i.p. injection in B6 mice (Figure 1A) with a significant decrease in the circulating monocytes (Figure 1B), granulocytes (Figure 1C) and lymphocytes (Figure 1D). No significant changes of body-weight was observed (data not shown) suggesting the absence of major CP-induced toxicity after a single dose. Furthermore, maximum neutropenia was observed at 3 days, and a significant recovery in neutrophil counts was found after 5 and 7 days post CP injection (Figure 1).

Next we established an acute pneumonia infection in immunocompromised B6 mice with *P. aeruginosa* for the evaluation of treatment effects of a single dose of the human monoclonal anti LPS serotype O11 IgM antibody, Panobacumab. Therefore, mice were submitted to mild, non-lethal (10^5^ cfu) and acute (10^6^ cfu) *P. aeruginosa* lung infection 3 days after CP injection at different doses in B6 mice. Bacterial load in the lung and lung weight, as a surrogate marker of pneumonia, were determined 24 h later. We observed a significant increase in lung bacterial load (Figure 1E) and lung weight (Figure 1F) after 100 mg/kg CP injected mice as compared to untreated controls, reaching approximately the same bacterial load in the lungs of untreated animals after an acute infection with a log higher inocoulum of 10^6^ cfu (Figure 1E and F). An additional increase of about 6-log in bacterial load was noticed after a treatment with 200 mg/kg CP, resulting in significant mortality rates (data not shown). In summary, an infection with *P. aeruginosa* strain 2310.55 with an inoculum of 10^5^ cfu 3 days after 100mg/kg CP resulted in a robust increase of lung weights and bacterial load indicating uncontrolled pneumonia. Therefore, these experimental parameters were chosen for the evaluation of the therapeutic Panobacumab efficacy.

### Therapeutic and specific effect of Panobacumab on *P. aeruginosa* infection in neutropenic mice

**Figure 2:**
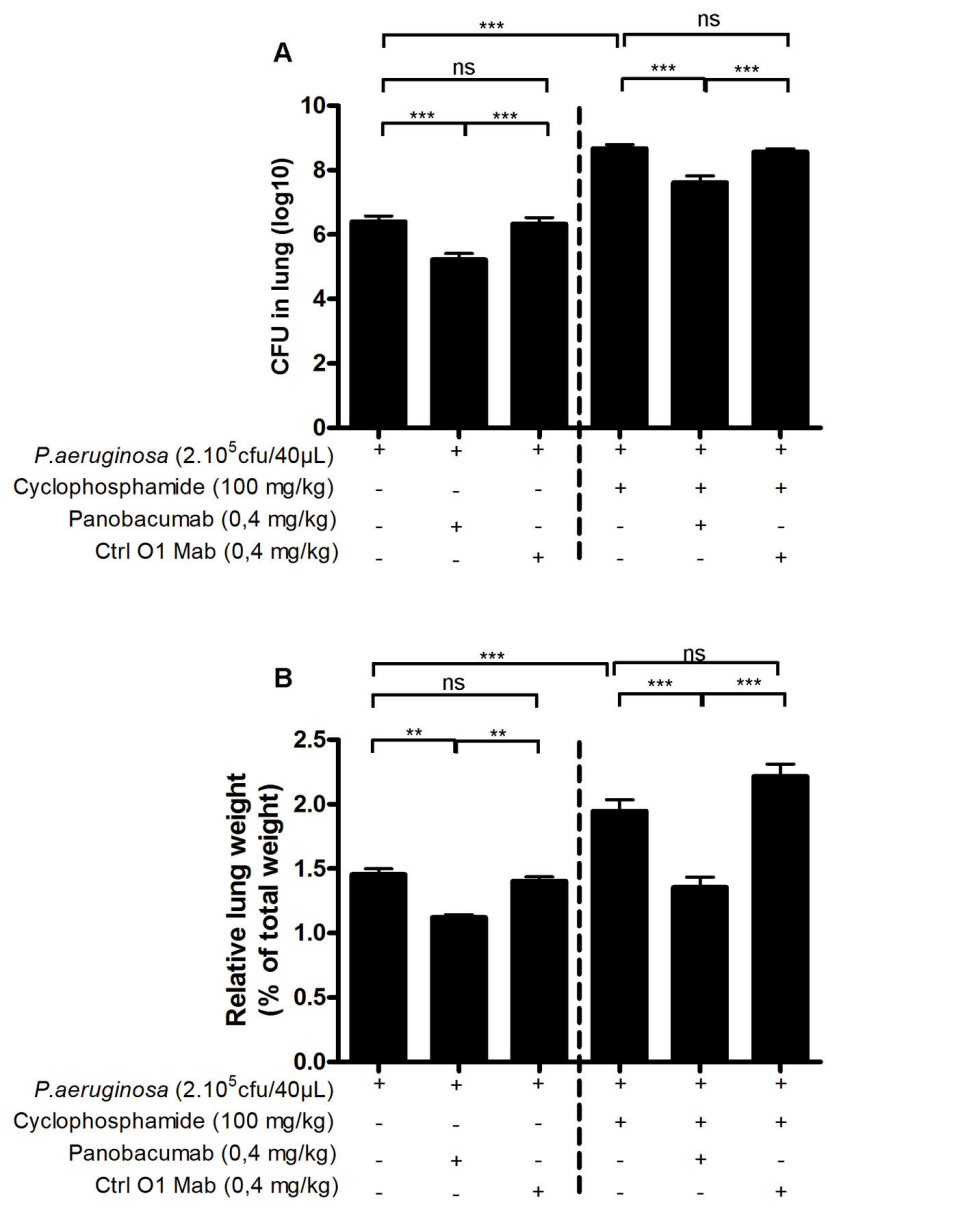
Therapeutic and specific effect of Panobacumab on *P. aeruginosa-*induced pneumonia in immunosuppressed mice. B6 mice were immunosuppressed with cyclophosphamide (100 mg/kg) and 3 days later infected by intra-nasal instillation of 40 µL of *P. aeruginosa* strain 2310.55 (2x10^5^ cfu). Panobacumab or a control anti-LPS: O1 IgM MAb (Ctrl O1 MAb, with specificity to serotype O1) were given i.v. at 0.4 mg/kg, 4 h after the infection. Lung cfu (A) and lung weight (B) were recorded 24 h after the infection. Groups of 14 mice were used and mean values ± SEM are shown (One-way ANOVA with Tukey’s Multiple Comparison Test; ns: non significant, * p<0.05, ** p<0.01, *** p<0.001). The results are a pool of two independent experiments.

**Figure 3:**
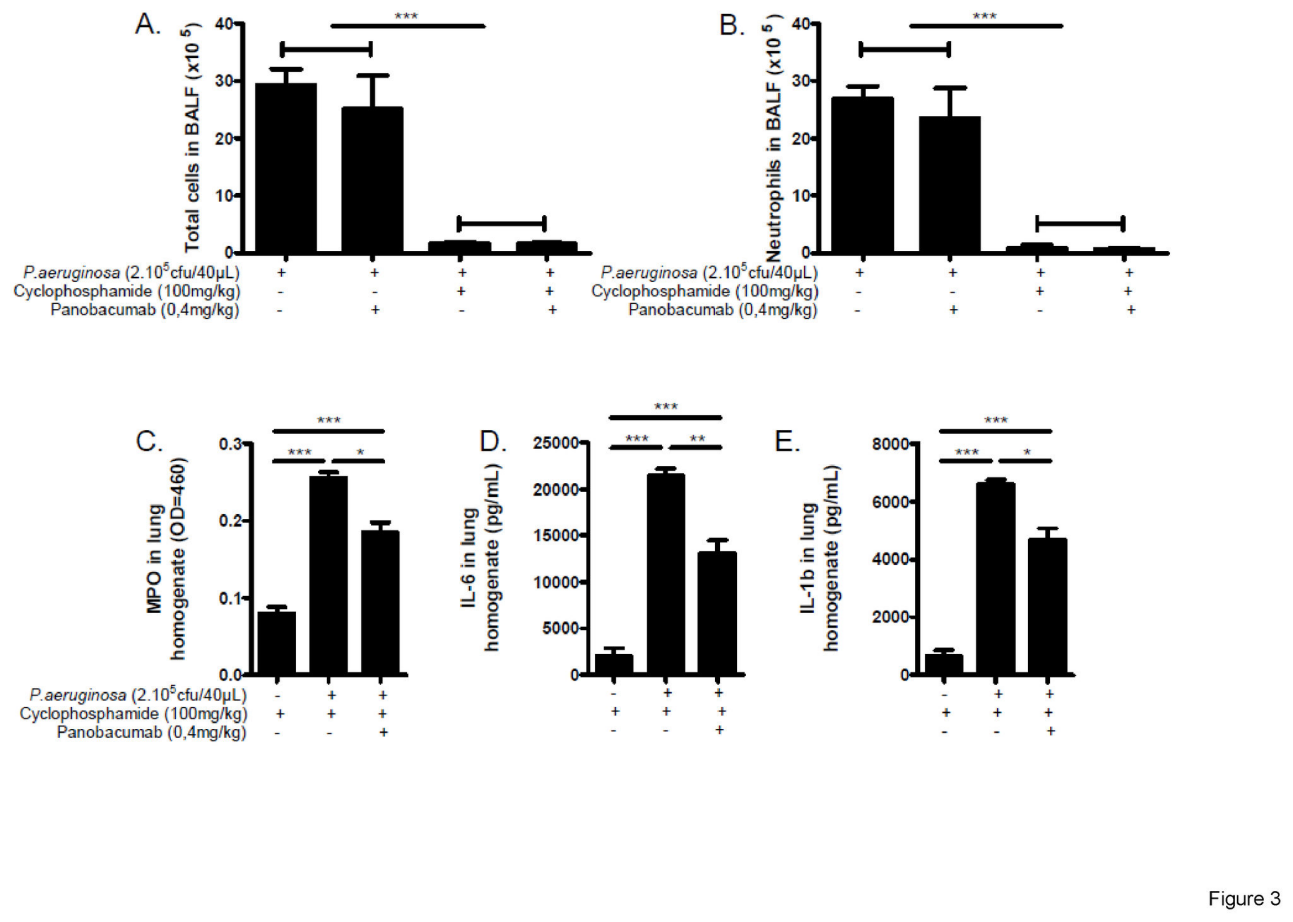
Reduced lung inflammation after Panobacumab treatment in immunosuppressed *P. aeruginosa-*infected mice. B6 mice were immunosuppressed with cyclophosphamide (100 mg/kg) and 3 days later infected by intra-nasal instillation of 40 µL of *P. aeruginosa* strain 2310.55 (2x10^5^ cfu). Panobacumab was given i.v. at 0.4 mg/kg, 4 h after the infection. Absolute numbers of cells (A), neutrophils (B) were measured in BALF 24 h after infection. Lung MPO (C) was recorded 24 h after infection. The concentrations of IL-6 (D) and IL-1β (E) in lung homogenates were determined 24 h after infection. Groups of 8-10 mice were used and mean values ± SEM are shown (One-way ANOVA with Tukey’s Multiple Comparison Test: * p<0.05, *** p<0.001). The results are a pool of two independent experiments.

**Figure 4:**
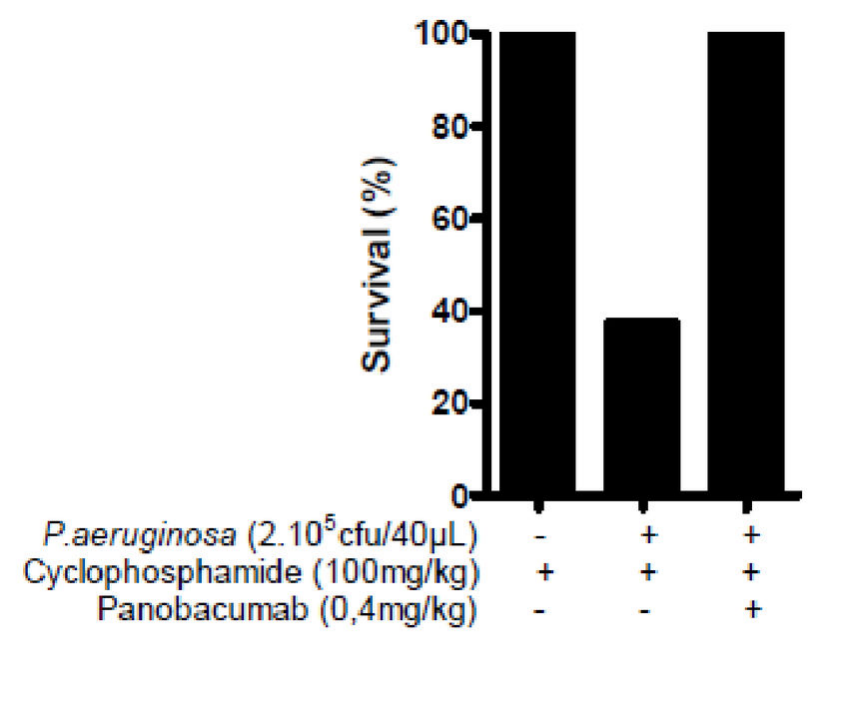
Enhanced survival after Panobacumab treatment in immunosuppressed *P. aeruginosa*-infected mice. B6 mice were immunosuppressed with cyclophosphamide (100 mg/kg) and 3 days later infected by intra-nasal instillation of 40 µL of *P. aeruginosa* strain 2310.55 (2x10^5^ cfu). Panobacumab was given i.v. at 0.4 mg/kg, 4 h after the infection. Survival was monitored 24 h after infection. Groups of 8-10 mice were used.

As previously demonstrated, passive immunization of animals with Panobacumab enhanced survival of animals after *P. aeruginosa* infection, reduced bacterial dissemination [17] and lung weight including pro-inflammatory cytokines and histological lesions in immunocompetent mice [18]. Since exacerbation of pneumonia is frequently observed in neutropenic patients, we tested in the animal model of neutropenia whether Panobacumab has a beneficial effect on *P. aeruginosa*-induced pneumonia by analyzing the bacterial load and lung weight as measure of lung inflammation. As expected, in immunocompetent mice, treatment of infected mice with 0.4 mg/kg Panobacumab resulted in significant decrease in bacterial load (Figure 2A) of about 1.2 cfu (log_10_) and lung weight (Figure 2B). Importantly, in animals with severely impaired immune system due to CP treatment, therapeutic treatment with a single dose of 0.4 mg/kg Panobacumab resulted in a comparable and significant reduction of bacterial load (Figure 2A) of and lung weight (Figure 2B) as compared to mice not treated with the antibody. We observed that upon CP treatment and after infection, inflammatory cell recruitment in BALF was greatly reduced (Figure 3A), reaching 90% diminution for neutrophils (Figure 3B), which is consistent with the systemic leucopenia we described in Figure 1; in which we do not observe any effect of Panobacumab on the recruited cell number. As we demonstrated a significant effect of Panobacumab treatment in bacterial clearance after infection (Figure 2), we investigated more specifically neutrophil recruitment in the lungs. Upon infection, in the immunosuppressed animals, Panobacumab induced a reduced neutrophil recruitment, as determined by MPO measurement, as a surrogate marker of neutrophil recruitment, in the lungs (Figure 3C). Inflammation is essential for host defense but can cause tissue damage and organ failure if exaggerated and protracted. Therefore we investigated the effect of local levels of inflammatory mediators upon *P. aeruginosa* infection in lung homogenates 24 h post-infection in immuno-compromised hosts. The pro-inflammatory cytokines IL-6 (Figure 3D) and IL-1β (Figure 3E) were significantly lower, after infection, in the lung compartment of panobacumab-treated animals as compared to untreated animals. This was accompanied by a significant survival benefit (Figure 4). These data clearly indicate that in the context of immunosuppression, therapeutic administration of Panobacumab, reduces significantly the production of inflammatory mediators. Finally, we investigated whether the protection effect could be mediated by the presence of nonspecific IgM antibodies e.g. having a broad anti-inflammatory effect. Therefore we included a monoclonal human IgM antibody directed against *P. aeruginosa* lipopolysaccharide serotype O1 as a control (Ctrl O1 Mab). In both, the immunocompetent and immunosuppressed context, no significant effect of the O1-specific antibody was observed (Figure 2). Collectively, these data demonstrate that a single dose of monoclonal antibody Panobacumab reduces, in a serotype specific manner, the bacterial load in the lung of severely neutropenic animals and thereby decrease inflammatory responses and pneumonia.

### Therapeutic and specific effect of Panobacumab on *P. aeruginosa* infection in combination with antibiotic administration

In the absence of strong adverse effects, current guidelines recommend the use of combination strategy of two anti-pseudomonal antibiotics when the local resistance patterns and patients risk factors suggest the opportunity of *P. aeruginosa* pneumonia. Combined therapy is thought to reduce the emergence of drug resistance [20] and to enhance the therapeutic due to synergy [21]. In this context, we investigated the combined effect of Panobacumab with a classical anti-pseudomonas antibiotic Meropenem.

**Figure 5:**
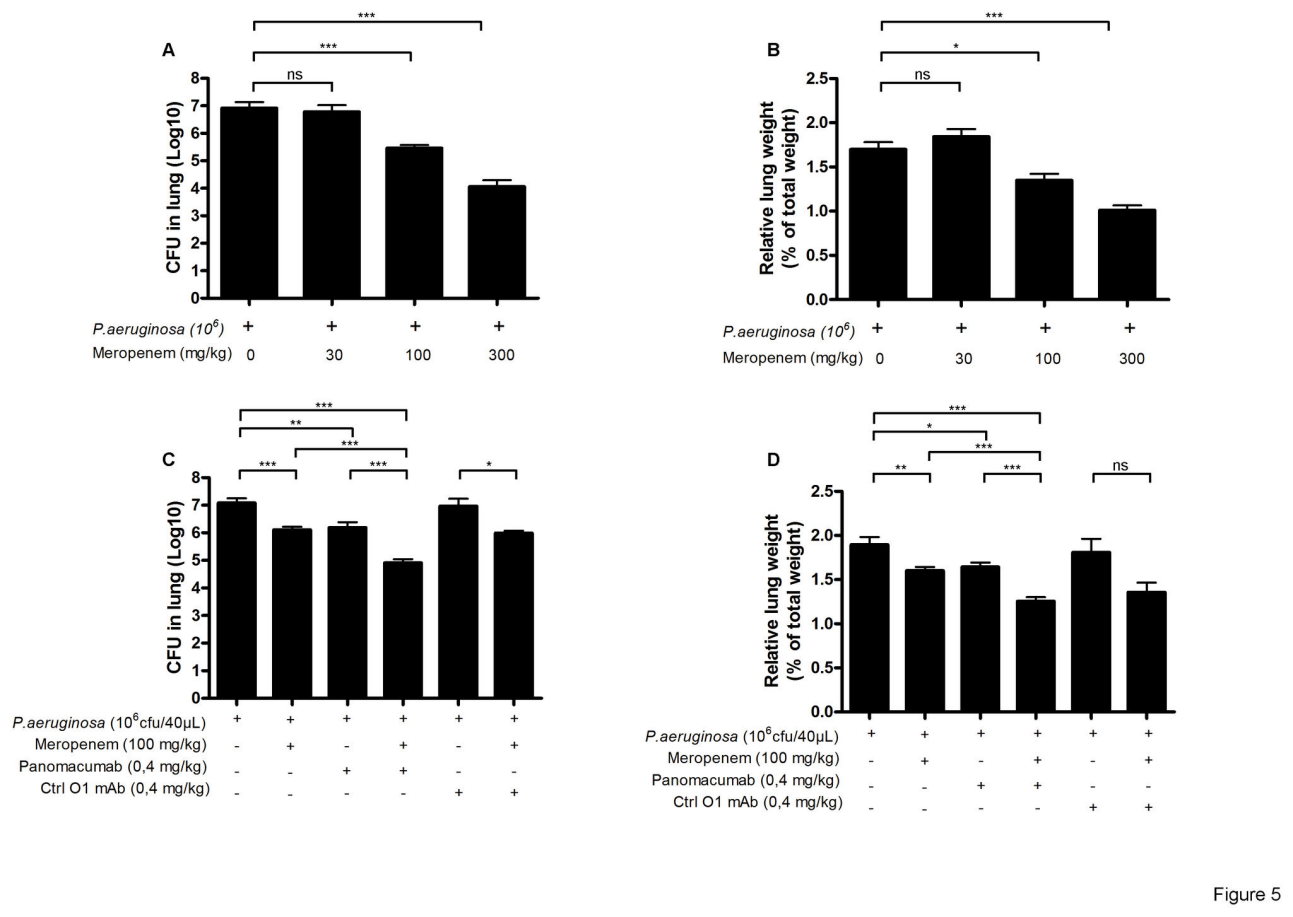
Effect of Panobacumab on *P. aeruginosa* infection alone and in combination with antibiotic administration. B6 mice received intra-nasal instillation of 40 µL of *P. aeruginosa* strain 2310.55 (10^6^ cfu). Meropenem or saline was given ip at 30, 100 and 300 mg/kg, 2 h after the infection. Lung cfu (A) and lung weights (B) were recorded 24 h after the infection. Panobacumab or a control anti-LPS: O1 IgM MAb (Ctrl O1 MAb, with specificity to serotype O1) were given i.v. at 0.4 mg/kg, 4 h after the infection, alone or in combination with Meropenem, lung cfu (C) and lung weight (D) and lung cfu (B) were recorded 24 h after the infection. Groups of 8-25 mice were used and mean values ± SEM are shown (One-way ANOVA with Tukey’s Multiple Comparison Test; ns: non significant, * p<0.05, ** p<0.01, *** p<0.001). The results are a pool of three independent experiments.

First, we titrated the amount of Meropenem in an acute infection with *P. aeruginosa* strain 2310.55. A dose of 30 mg/kg was ineffective, whereas doses of 100 and 300 mg/kg induced a significant reduction in both bacterial load as well as lung weight (Figure 5A and B). For further experiments in the combination studies, the dose of 100 mg/kg of Meropenem was chosen, approximately corresponding to a scale-down of dose used in humans for 72h of treatment [22]. Meropenem treatment was started 2 hours and Panobacumab was given 4 hours after the infection. We selected this scenario to model the clinical situation where antibiotics treatment is generally started immediately after a suspected pneumonia while a specific immunotherapy would be initiated at a later time point after confirmation of a *P. aeruginosa* infection. Upon infection, Panobacumab-treated mice exhibited reduced bacterial load (Figure 5C) and lung weight (Figure 5D) as compared to untreated or control IgM antibody treated animals. In addition, Meropenem-treated animals presented a mild attenuation of lung weight. Nevertheless, the combined treatment of Meropenem and Panobacumab lead to an additive reduction of bacterial load and lung weight when compared to single-treated groups (Figure 5C and D). Therefore the results clearly indicate a beneficial effect of using Panobacumab in combination with antibiotics for the treatment of *P. aeruginosa* pneumonia.

### Therapeutic effect of Panobacumab on antibiotic resistant *P. aeruginosa* infection

*P. aeruginosa* is a common multidrug-resistant (MDR) gram-negative agent causing pneumonia in hospitalized patients [7]. In this context, we investigated the effect of Panobacumab against a Meropenem-resistant *P. aeruginosa* strain.

**Figure 6:**
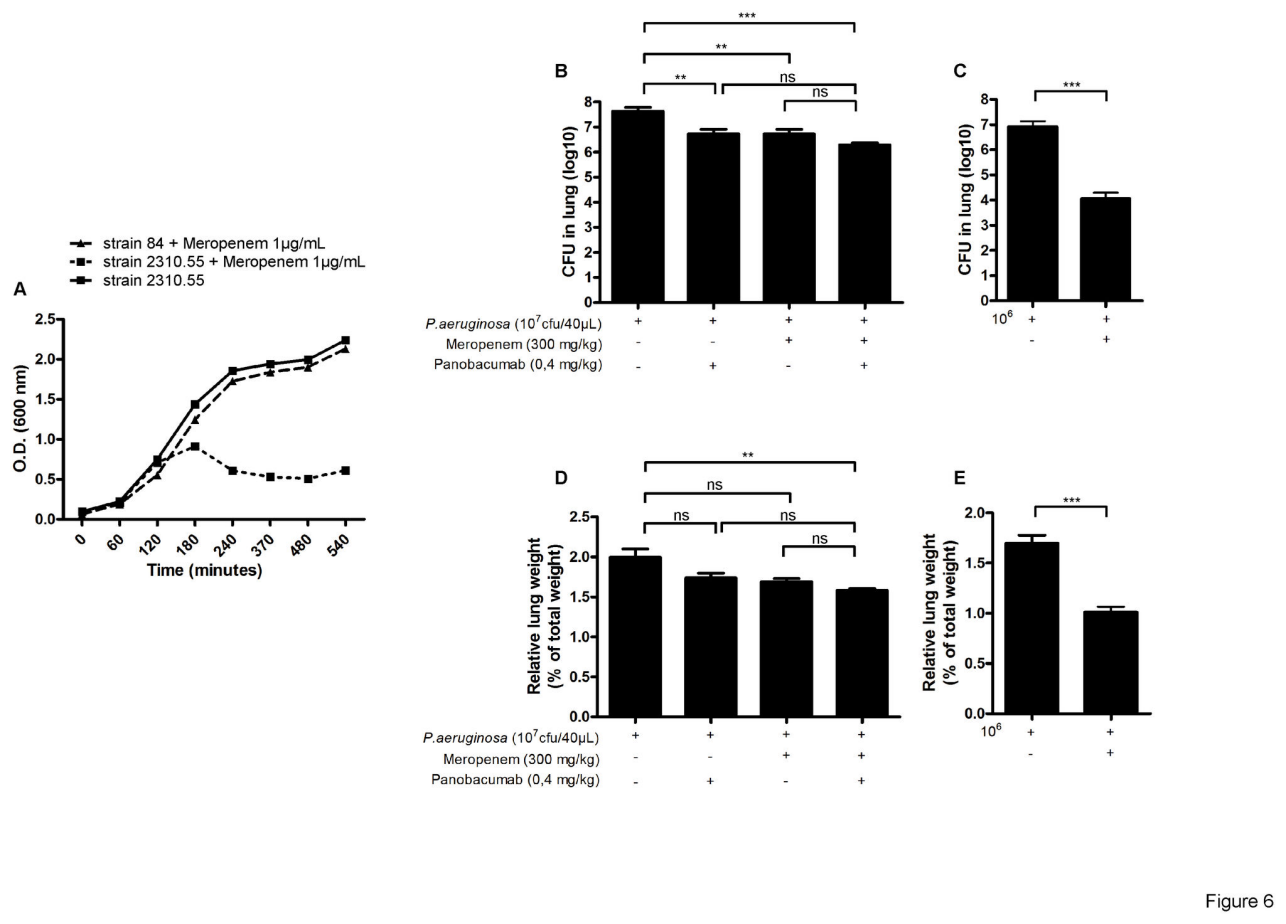
Effect of Panobacumab on Meropenem-resistant *P. aeruginosa* infection. After overnight preculture, *P. aeruginosa* strain 84 and 2310.55 were grew for 9 h in the presence or not of 1µg/mL of Meropenem. Optical density at 600nm was recorded over the period (A). B6 mice received intra-nasal instillation of 40 µL of Meropenem-resistant *P. aeruginosa* strain 84 (10^7^ cfu) (B and D) or classical strain 2310.55 (10^6^ cfu) (C and E). Meropenem or saline was given ip at 300 mg/kg, 2 h after the infection. Panobacumab was given i.v. at 0.4 mg/kg, 4 h after the infection, alone or in combination with Meropenem. Lung cfu (B and C) and lung weights (D and E) were recorded 24 h after the infection. Groups of 7 mice were used and mean values ±SEM are shown (One-way ANOVA with Tukey’s Multiple Comparison Test; ns: non significant, * p<0.05, ** p<0.01, *** p<0.001). The results are a pool of two independent experiments.

We first validated the Meropenem resistance of strain 84 *in vitro* as compared to the classical non-resistant strain 2310.55. In the presence of 1µg/mL of Meropenem in the culture broth, strain 2310.55 was unable to grow as compared to strain 84 which grew comparable to the untreated strain 2310.55 (Figure 6A). We then addressed the effect of Panobacumab against an acute lung infection with *P. aeruginosa* strain 84. A high dose of Meropenem (300mg/kg) was given 2 hours after the infection while Panobacumab treatment started 4 hours after the infection. Upon infection, Panobacumab-treated animals exhibited a significant decrease of lung bacterial load (Figure 6B) as compared to untreated animals. Meropenem-treated animals presented a similar slight reduction of lung infection (about 1log) which heavily diminished compared to the one observed with the non-resistant *P. aeruginosa* strain 2310.55 where Meropenem induced a large reduction in both lung bacterial load (Figure 6C) and lung weight (Figure 6E). In the context of Meropenem-resistant *P. aeruginosa* acute lung infection, the combined treatment of Meropenem and Panobacumab did not lead to an additive reduction of lung parameters (Figure 6B and D) as compared to combined treatment against non-resistant *P. aeruginosa* strain (Figure 6C and D). In summary, our results demonstrated a beneficial effect in treatment of *P. aeruginosa* pneumonia with Panobacumab independently of the resistance profile of the infecting *P. aeruginosa* strain.

## Discussion

Previously we established in immunocompetent animals a clear benefit for the treatment of *P. aeruginosa* (serotype O11) pneumonia with Panobacumab leading to a reduction in bacterial load in the lungs and enhanced survival benefit [18]. We reported here that therapeutic administration of Panobacumab protects neutropenic mice from acute pneumonia. To evaluate the efficacy of this antibody in a clinical situation mimicking immunocompromised patients, we first established a model of neutropenia using cyclophosphamide (CP), a nitrogen alkylating molecule used as anti-cancerous agent in the clinic [23]. Once injected intraperitoneally, CP resulted in a 60-95% reduction of leukocyte numbers depending on the CP dose, including a massive reduction of granulocytes in whole blood after three days, indicating a severely immunocompromised state. Using this model, we showed that CP-treated mice exhibited a high susceptibility to *P. aeruginosa* infection associated with severe pneumonia and increased bacterial load This is in line with earlier publications reporting enhanced susceptibility to *P. aeruginosa* in CP-immunosuppressed mice [24]. Once established, we investigated the effect of Panobacumab on bacterial clearance and pneumonia induced by *P. aeruginosa* in this immunocompromised mouse model, choosing a regimen of CP dosing that allowed to achieve a significant immune suppression without an increase in mortality. We showed that systemic administration of Panobacumab induced a significant decrease in lung weight and bacterial load, leading us to the conclusion that the observed effects were dependent on the interaction between Panobacumab and O11-LPS from the infecting *P. aeruginosa* strain, as no positive effect was noticed against an O1-LPS strain. In the CP-induced neutropenia model (100mg/kg), the number of granulocytes was reduced about 80% as compared to untreated mice, which is similar to the observed massive leucopenia present in hematopoietic transplantation recipient [25,26] or HIV patients [27]. Despite severe immune suppression, the treatment with Panobacumab resulted in a reduction of bacterial load and inflammation in the lung, indicating that reduced leukocyte numbers might still be sufficient to mediate bacterial clearance after opsonization by Panobacumab. The first immune cells encountering *P. aeruginosa* in the lung are alveolar macrophages which can internalize and eliminate bacterial pathogens. However, their main functions probably rely on the production of chemokines and cytokines, which are then responsible for neutrophil recruitment [28,29]. In fact, depletion of macrophages has been reported to result in contradictory outcomes regarding bacterial clearance, suggesting that the presence of very few macrophages could be sufficient for induction of neutrophil recruitment [30–32]. Neutrophil recruitment to the lung is a major component of the protective host immune response against *P. aeruginosa* pneumonia, as neutrophil depletion renders mice highly susceptible to *P. aeruginosa* infections [33,34]. We have shown previously that treatment with Panobacumab led to a massive accumulation of granulocytes in broncho-alveolar lavage and in the lungs [18]; and the proposed modes of action include antibody–mediated opsonophagocytosis and complement activation. Thus phagocytes, such as granulocytes and macrophages, constitute an integral part of the mechanism of action of Panobacumab. As such, a beneficial effect of this treatment in a situation of profound leucopenia was not readily observed. Alternatively phagocyte-independent effects, such as immobilization of bacteria, or complement activation could also contribute to bacterial clearance in this model. Both effectors functions have been shown to play a critical role in the activity of antibodies against bacterial cell surface components, including antibodies to *P. aeruginosa* lipopolysaccharide [35]. In addition the reduced lung levels of IL-6, IL-1β as well as neutrophil influx upon infection under Panobacumab treatment would indicate that the reduction of the bacterial load by Panobacumab resulted in an overall attenuated pro-inflammatory response. Unrestrained up-regulation of the inflammatory response is associated with exacerbated tissue injury and severe airway disease.

In the clinic, antibiotics are administered immediately after confirmation of bacterial infection. Nevertheless, therapeutic strategies have evolved following a “hit hard and hit fast” rule to provide an initial and critical adequate therapy, which is associated with reduced morbidity and mortality. Recent guidelines for pneumonia management recommended combined use of anti-pseudomonal agents [36]. Meropenem is a widely used antibiotic with high activity against most gram-negative bacteria and it is commonly used in empiric therapies for infectious diseases [37]. Therefore patients receiving Panobacumab for the treatment of pneumonia will always receive antibiotics before the diagnosis of a *P. aeruginosa* infection has been established. Thus, in the co-treatment model described here, mice were treated with Meropenem 2 hours after challenge and after additional 2 hours with Panobacumab to mimic clinically relevant treatment schedules. Meropenem and Panobacumab treatments alone resulted in a reduction of bacterial load and inflammation in the lung, but Panobacumab had an additive effect in the co-treatment experiments. Similar effects have been described for a nontherapeutical treatment with anti-PcrV (Mab166) and Tobramycin [38]. Nevertheless, as we have administered the treatment after the infection, we could demonstrate a therapeutic benefit for the combination of Panobacumab with Meropenem administration, providing evidence for a new, more effective therapeutic strategy against *P. aeruginosa* infections. Recent reports have established that nosocomial strains of *P. aeruginosa* are frequently multi-drug resistant and that their prevalence is increasing worldwide, especially in high-risk populations [39,40]. Clinical treatment schedule for suspected pneumonia always include antibiotics and especially Meropenem which is the prototypal anti-pseudomonas antibiotic. Upon pneumonia induced by Meropenem-resistant *P. aeruginosa* strain, this treatment should be ineffective and lead to deteriorated patient condition. In this context of a diminished host response, such as immunosuppression induced by cyclophosphamide-treatment, we could demonstrate here that the therapeutic administration of Panobacumab therapy significantly reduced lung bacterial load in mice infected with Meropenem-resistant *P. aeruginosa* strain. Surprisingly Meropenem at 300mg/kg still had an effect as compared to the untreated control group, despite that the strain was identified *in vitro* as resistant. In this context it has to be noted that the virulence of this isolate was significantly smaller than the sensitive strain 2310.55 used, as seen by the higher inoculum necessary to establish an infection (10^7^ as compared to 10^6^). Therefore it is possible that the addition of a high dose of Meropenem simply slowed bacterial growth, resulting in a significant difference in bacterial load after 24 hours compared to untreated animals. Furthermore, the reduction in bacterial load of approx. 1 log in 24 hours upon treatment with Panobacumab was in line with results seen with the Meropenem sensitive strain, and addition of a high dose of Meropenem in combination with Panobacumab did not improve the effects, which indicates again that the effect observed was due to the use of Panobacumab. In conclusion, our study provides evidence for a beneficial effect of Panobacumab therapy for *P. aeruginosa* pneumonia in a model of severe neutropenia as well as in a combination therapy with Meropenem. Furthermore Panobacumab therapy also showed efficacy against one meropenem resistant *P. aeruginosa* strain, which is an important step in the light of the fact that most severe nosocomial infections with *P. aeruginosa* occur in immunocompromised patients and that emergence of antibiotic resistant strain poses a significant risk to standard antibiotic therapy. Overall the results presented here could open the door for the inclusion of immunosuppressed patients with resistant infections in clinical studies of antibody treatment.
